# Supplementary figures and images for: The presence of Wormian bones increases the fracture resistance of equine cranial bone
Source: PLoS One. 2021 Apr 16;16(4):e0249451. doi: 10.1371/journal.pone.0249451 (PMC8051753; doi:10.1371/journal.pone.0249451)

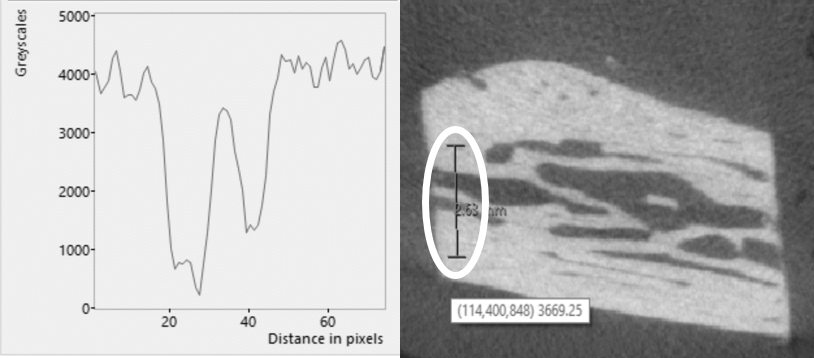

Supplement: S1 Fig — (TIF) [file pone.0249451.s001.tif]

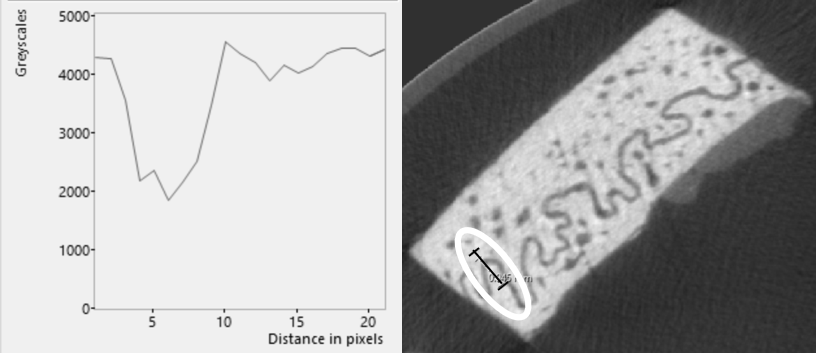

Supplement: S2 Fig — (TIF) [file pone.0249451.s002.tif]
