# Supplementary material for: The presence of Wormian bones increases the fracture resistance of equine cranial bone
Source: PLoS One. 2021 Apr 16;16(4):e0249451. doi: 10.1371/journal.pone.0249451 (PMC8051753; doi:10.1371/journal.pone.0249451)
Supplement: S3 Fig — (PDF) [file pone.0249451.s003.pdf]

Sample	Horse	Orientation	Sex	Age	Wormian	Span (mm)	Av. width (mm)	Av.thickness (mm)	Displacement (mm)	P max (N)	Stiffness (N/mm)	BMD (mg/cc)	Stiffness normalised (N/mm^2)	E (Mpa)
121 L1-2-2	121	L	Male	29	Non wormian	38.16	11.76	4.09	0.98	159.79	182.22	946.38	3.79	3146.18
121 L2-1	121	L	Male	29	Non wormian	35.58	8.55	4.32	1.12	172.99	173	914.06	4.68	2824.99
121 L2-3-2	121	L	Male	29	Non wormian	37.28	9.34	4.56	0.81	141.23	183.16	879.03	4.30	2672.08
121 T1	121	T	Male	29	Non wormian	49.1	7.73	9.46	2.25	320.07	150.15	1117.43	2.05	679.70
121 T2	121	T	Male	29	Non wormian	46.22	9.23	5.75	1.23	164.38	147.69	819.70	2.78	2073.31
121 T3	121	T	Male	29	Non wormian	40.87	9.10	7.07	0.90	134.60	161.37	900.62	2.51	854.88
122 L1	122	L	Female	22	Non wormian	40.54	8.55	5.55	1.47	237.05	205.93	949.70	4.34	2350.06
122 L2-2	122	L	Female	22	Non wormian	40.25	7.72	4.79	1.69	187.61	136.2	960.08	3.68	2610.34
122 L3	122	L	Female	22	Non wormian	55.5	12.72	5.92	1.31	304.72	245.12	936.38	3.26	3977.34
123 L1	123	L	Male	17	Non wormian	36.1	7.28	5.28	2.05	249.83	138.33	845.95	3.60	1518.95
124 L1	124	L	Female	14	Non wormian	52.23	8.45	4.14	1.17	142.62	135.66	1044.17	3.88	5550.17
124 L2	124	L	Female	14	Non wormian	46.78	8.33	4.06	1.49	140.23	104.32	1117.79	3.08	3672.38
124 L3	124	L	Female	14	Non wormian	43.44	12.27	5.90	0.89	223.81	268.18	1145.36	3.71	2579.06
124 L4	124	L	Female	14	Non wormian	44.78	10.86	5.09	1.72	141.14	100.75	1135.64	1.82	1736.02
124 T1	124	T	Female	14	Non wormian	32.76	10.39	2.35	1.72	64.52	43.102	994.13	1.76	2847.04
124 T2	124	T	Female	14	Non wormian	33.73	9.86	3.36	1.10	59.31	70.587	1163.35	2.13	1842.58
124 T3	124	T	Female	14	Non wormian	40.63	10.21	3.79	1.40	55.29	48.015	1107.35	1.24	1617.33
171 L1	171	L	Female	10	Non wormian	40.75	9.66	5.59	1.34	239.47	247.65	957.92	4.58	2944.82
171 L2	171	L	Female	10	Non wormian	38.9	9.12	5.68	2.00	520.38	320.43	880.81	6.18	2206.78
171 L3	171	L	Female	10	Non wormian	43.12	8.13	5.65	1.01	170.19	186.82	1075.97	4.06	1792.78
171 L4	171	L	Female	10	Non wormian	38.8	11.64	5.68	1.49	267.16	212.27	975.09	3.21	2885.09
172 L2	172	L	Female	4	Non wormian	42.49	5.87	5.25	0.85	117.63	255.35	1263.74	8.28	2813.93
172 L3	172	L	Female	4	Non wormian	60.66	12.26	5.39	1.29	108.57	100.56	999.16	1.52	4038.41
173 L1	173	L	Female	7	Non wormian	36.77	7.75	4.12	1.40	196.40	174.82	1141.75	5.47	3962.82
173 L2	173	L	Female	7	Non wormian	39.4	7.01	6.26	1.53	207.24	195.34	1514.55	4.45	1236.24
173 L3	173	L	Female	7	Non wormian	38.4	7.84	4.37	1.48	242.08	173.03	1128.66	5.05	2520.79
173 T2	173	T	Female	7	Non wormian	46.95	9.83	4.56	1.16	105.91	109.65	734.01	2.44	3298.83
174 L2	174	L	Female	27	Non wormian	40.75	11.62	6.30	1.62	266.14	177.98	817.24	2.43	1682.99
174 T1	174	T	Female	27	Non wormian	49.26	9.17	5.49	1.60	370.00	318.3	1359.08	6.32	5414.13
174 T2	174	T	Female	27	Non wormian	51.63	9.05	8.44	1.46	89.67	92.415	1201.84	1.21	521.48
174 T3	174	T	Female	27	Non wormian	45.88	7.32	5.81	0.78	248.73	368.1	1009.41	8.65	4538.90
H1-T2	1	T	Male	18	Non wormian	35.67	8.75	4.25	0.79	68.10	98.622	946.05	2.65	1666.53
H1-L1	1	L	Male	18	Non wormian	40.41	11.65	4.44	0.82	256.86	377.45	1009.94	7.30	6118.51
H1-L3	1	L	Male	18	Non wormian	36.53	9.96	4.15	1.48	146.59	127.43	680.98	3.08	2181.51
121 L1-1	121	L	Male	29	Wormian	36.36	10.34	4.12	1.43	192.79	164.24	1002.55	3.85	2721.98
121 L1-3	121	L	Male	29	Wormian	35.11	10.65	5.89	1.58	283.07	213.92	944.46	3.41	1062.15
121 L2-2	121	L	Male	29	Wormian	38.12	9.50	5.84	0.95	208.06	257.28	1096.99	4.64	1885.53
122 T1	122	T	Female	22	Wormian	45	8.84	5.64	0.92	108.84	135.24	1029.49	2.71	1941.91
122 T2	122	T	Female	22	Wormian	33.74	9.37	4.13	1.56	158.20	126.88	1081.65	3.28	1841.31
122 T3	122	T	Female	22	Wormian	32.9	9.16	4.68	1.05	79.26	92.68	1081.65	2.16	878.46
123 T1-2	123	T	Male	17	Wormian	41.23	7.70	4.29	2.28	55.23	25.865	780.92	0.78	680.62
123 T2	123	T	Male	17	Wormian	40	8.21	3.35	2.58	61.29	31.014	809.27	1.13	1579.80
172 L1	172	L	Female	4	Wormian	55.91	11.56	5.05	1.14	187.46	175.63	1048.27	3.01	4860.07
172 T1	172	T	Female	4	Wormian	51.36	12.03	4.10	1.96	121.93	71.384	943.12	1.45	3112.96
172 T2	172	T	Female	4	Wormian	46.7	8.89	3.79	2.14	83.91	49.886	1116.97	1.48	2164.38
172 T3	172	T	Female	4	Wormian	45.29	11.30	3.38	3.00	91.57	48.061	1044.68	1.26	3728.22
172 T4	172	T	Female	4	Wormian	49.6	10.78	3.87	1.86	40.00	21.739	960.79	0.52	1628.77
H1-T1	1	T	Male	18	Wormian	35.67	9.69	3.65	2.41	213.88	98.78	949.09	2.80	2385.10
H1-T3	1	T	Male	18	Wormian	35.67	9.38	3.92	1.77	106.69	61.909	896.46	1.68	1239.60
H1-T4	1	T	Male	18	Wormian	32.53	8.50	3.49	1.77	115.66	95.042	760.50	3.20	2263.67
H1-L2	1	L	Male	18	Wormian	40.41	10.68	4.46	0.92	168.86	198.33	1264.56	4.16	3445.45
H2-L1	2	L	Female	18	Wormian	36.8	7.82	7.14	0.74	286.47	471.06	902.50	8.44	2062.74
H2-L2	2	L	Female	18	Wormian	36.8	8.56	4.71	1.58	128.61	105.84	858.09	2.63	1474.91
H2-L3	2	L	Female	18	Wormian	36.8	8.74	6.58	0.82	256.58	352.42	732.67	6.13	1762.74
H2-L4	2	L	Female	18	Wormian	36.8	8.53	6.20	2.20	328.94	250.5	887.52	4.74	1537.69
H2-T3	2	T	Female	18	Wormian	41.33	8.81	5.55	1.23	198.14	198.08	1313.12	4.05	2321.25
