# Supplementary material for: The presence of Wormian bones increases the fracture resistance of equine cranial bone
Source: PLoS One. 2021 Apr 16;16(4):e0249451. doi: 10.1371/journal.pone.0249451 (PMC8051753; doi:10.1371/journal.pone.0249451)
Supplement: Attachment — Submitted filename: response to reviewers.pdf [file pone.0249451.s004.pdf]

UCD School of Mechanical and Materials Engineering

Scoil na hInnealtóireachta Meicniúla agus Ábhar UCD

University College Dublin,
Belfield, Dublin 4, Ireland

An Coláiste Ollscoile Baile Átha Cliath,
Belfield, Baile Átha Cliath 4, Eire

T +353 1 716 1889
F +353 1 283 0534

aisling.niannaidh@ucd.ie
www.ucd.ie/eacollege/mme/

12th Oct 2020

Re: The presence of Wormian bones increases the fracture resistance of equine cranial bone

Dear Editor,

The authors submitted a previous manuscript on a related topic to Plos One (Pone-D-19-04273) in which a major revision was advised. This decision was arrived at while I was on maternity leave in 2019. On the advice of the editor, the current manuscript has been reanalysed, refocussed and completely rewritten. To highlight how the current manuscript has been revised considering the original review, I have included a high level rebuttal (given that the manuscript has undergone a significant change) addressing the original reviewer's concerns.

Reviewer 3:

Reviewer 3's primary concern centred around the fact that the purpose of creating an idealised FE model was unclear and was unjustified. Furthermore, the reviewer felt that the description of the model was insufficient.

The justification for the idealised models is now more clearly laid out at the beginning of section 'numerical simulations' (L201-L207). Furthermore, rewording of statements in the abstract, introduction, methods and discussion sections now lends itself to a more coherent justification of the use of an idealised FE model.

Reviewer 4:

Reviewer 4 felt that presentation of the data in the paper was not optimal. They voiced concerns about the statistical methods applied and suggested a separate statistics section. They also advised a separate results and discussion section.

The results and statistics sections have undergone major changes. The analysis of specimen orientation, sample deflection and strain rate has been removed, leading to a

UCD School of Mechanical and Materials Engineering

Scoil na hInnealtóireachta Meicniúla agus Ábhar UCD

University College Dublin,
Belfield, Dublin 4, Ireland

An Coláiste Ollscoile Baile Átha Cliath,
Belfield, Baile Átha Cliath 4, Eire

T +353 1 716 1889
F +353 1 283 0534

aisling.niannaidh@ucd.ie
www.ucd.ie/eacollege/mme/

clearer presentation of the most relevant data. The results and discussion sections were separated (as recommended) and there was a significant rewrite of the discussion section.

Given the limited number of subjects, the reviewer criticised the analysis of effect of sex and age on horses.

The analysis is still included but now with the admission that a larger number of samples is required. A graph indicating sex, age and presence of Wormian bone (as recommended) is also now included in Fig. 7.

The general impression of the original reviewers seemed to be that the original manuscript represented novel and interesting findings, but that in its original format, the presentation of data and interpretation of the results were unclear. In this current manuscript these findings remain novel and interesting, however the presentation has been carefully revised so that the results more clearly articulated.

Kind regards,

Dr. Aisling Ní Annaidh
Associate Professor
School of Mechanical and Materials Engineering
University College Dublin
