# Supplementary material for: The presence of Wormian bones increases the fracture resistance of equine cranial bone
Source: PLoS One. 2021 Apr 16;16(4):e0249451. doi: 10.1371/journal.pone.0249451 (PMC8051753; doi:10.1371/journal.pone.0249451)
Supplement: Attachment — Submitted filename: response to the reviewers.pdf [file pone.0249451.s005.pdf]

UCD School of Mechanical and Materials Engineering

Scoil na hInnealtóireachta Meicniúla agus Ábhar UCD

University College Dublin,
Belfield, Dublin 4, Ireland

An Coláiste Ollscoile Baile Átha Cliath,
Belfield, Baile Átha Cliath 4, Eire

T +353 1 716 1889
F +353 1 283 0534

aisling.niannaidh@ucd.ie
www.ucd.ie/eacollege/mme/

9th Feb 2021

Re: The presence of Wormian bones increases the fracture resistance of equine cranial bone – response to reviewers

Dear Editor,

The authors would like to thank the reviewers for their helpful and insightful comments, questions and suggestions. The authors believe we have fully addressed all concerns and queries and that the manuscript is improved as a result.

An itemised response to each reviewer question / comment is given in blue text below. Changes which have been made to the manuscript are shown in red in the document 'manuscript v2_marked up' and the corresponding line numbers are provided. Sentences which have been deleted in the manuscript have been strikedthrough and are also in red. The fully revised manuscript (without markings) is provided in the document 'manuscript v2'.

Reviewers Comments:

Reviewer #1: This manuscript is an interesting examination of the presence and function of Wormian or inter-sutural bones in the horse skull. While considerable attention has been devoted to such bones in humans, little work has been directed at the extent to which Wormian bones are observed in other mammalian taxa. What's particularly nice about this manuscript is that the authors use microCT to characterize morphological variation in Wormian bones and then complement such data with tests of the mechanical properties of the bony elements. Thus, my comments and criticism are largely restricted to the need to tie in the current findings of this study into a larger body of research on the biomechanics and function of the neurocranium.

UCD School of Mechanical and Materials Engineering

Scoil na hInnealtóireachta Meicniúla agus Ábhar UCD

University College Dublin,
Belfield, Dublin 4, Ireland

An Coláiste Ollscoile Baile Átha Cliath,
Belfield, Baile Átha Cliath 4, Eire

T +353 1 716 1889
F +353 1 283 0534

aisling.niannaidh@ucd.ie
www.ucd.ie/eacollege/mme/

Major Comments:

Unlike other regions of the skeleton such as the limbs and jaws, the neurocranium in mammals is characterized by low levels of in vivo bone strain as well as the absence of load-induced bone formation or dietary plasticity (e.g., Hylander et al., 1991, Am. J. Phys. Anthropol.; Hylander & Johnson, 1997, Am. J. Phys. Anthropol.; Ravosa et al., 2010, Anat. Record; Franks et al., 2016, Anat. Record; Franks et al., 2017, Zoology). Whereas safety factors to failure are uniform for mammalian jaws vis-a-vis masticatory stresses (much as the case for limb elements and locomotor stresses), the circumorbital region and calvarium are considerably overbuilt to resist routine biting/chewing stresses (Ravosa et al., 2010). Hylander and colleagues have argued that neurocranial bones are designed to counteract infrequent, traumatic loads that could otherwise inflict damage to the underlying brain. Accordingly, bone formation in the neurocranium may be buffered to variation environmental/mechanical stimuli so as to ensure that sufficient bone exists to resist traumatic loads (rather than masticatory forces) (Ravosa & Kane, 2017, Zoology). Thus, the authors' current findings regarding the greater compliance to external stresses afforded by Wormian bones is wholly consistent with the evidence from prior in vivo bone strain and adaptive plasticity work in mammalian crania. This, as well as the similar presence of diploe throughout neurocranial bones in humans, is yet another potential design feature that may exist to dissipate traumatic cranial loads that might otherwise injure subjacent neural tissues. Arguably, this paper would be strengthened significantly if the authors were to link their findings to this larger body of biological research on craniofacial design and biomechanics.

An additional paragraph (L100-L109) has been added to the introduction incorporating some of the points and references outlined by reviewer 1. Additionally, the authors have included a stronger statement at the end of the

UCD School of Mechanical and Materials Engineering

Scoil na hInnealtóireachta Meicniúla agus Ábhar UCD

University College Dublin,
Belfield, Dublin 4, Ireland

An Coláiste Ollscoile Baile Átha Cliath,
Belfield, Baile Átha Cliath 4, Eire

T +353 1 716 1889
F +353 1 283 0534

aisling.niannaidh@ucd.ie
www.ucd.ie/eacollege/mme/

introduction suggesting that the presence of Wormian bones may act as "an additional design feature that may exist to dissipate traumatic cranial loads and protect the underlying brain" (L114).

Minor Comments:

The authors observations about the presence of sandwich bone or diploe should be related to the similar presence in humans of hard-tissue organization where cortical bone along the periosteal and endosteal surfaces has intervening diploe or spongy bone in the neurocranium.

An additional sentence (L362) has been added to the discussion.

There appear to be formatting inconsistencies in the references where article titles sometimes capitalize all of the nouns and sometimes not. There's also some variability in journal citation formatting.

The captalisation of nouns in some of the references has been changed and the citation formatting has been homogenised.

Reviewer #2: In general, this article is a well-executed study of the biomechanical effects of Wormian bones on biomechanical properties of cortical vault bone. Usually Wormian bones are analyzed as a non-metric skeletal trait with a genetic background. Because their prevalence varies across populations, analysis in humans specifically focuses on their presence, absence, or number as a phylogenetic signature. This paper is interesting in that it instead focuses on their contribution to cranial mechanical integrity. The finding that Wormian bones can reduce the risk of mechanical failure is novel and significant. This has implications for studying the evolutionary origin and prevalence of Wormian bones in different species.

UCD School of Mechanical and Materials Engineering

Scoil na hInnealtóireachta Meicniúla agus Ábhar UCD

University College Dublin,
Belfield, Dublin 4, Ireland

An Coláiste Ollscoile Baile Átha Cliath,
Belfield, Baile Átha Cliath 4, Eire

T +353 1 716 1889
F +353 1 283 0534

aisling.niannaidh@ucd.ie
www.ucd.ie/eacollege/mme/

Lines 45-74: This introduction, while thorough, is overly long and extraneous to the matter at hand. I would suggest condensing it to focus specifically only on the process that produces Wormian bones.

Lines 57-77 (three paragraphs) have been removed from the introduction. In response to comments from reviewer 1, an additional paragraph has been added (see above).

Lines 75-96: This is an appropriate level of introduction to the topic if combined with a condensed version of the preceding paragraphs. The justification for the study is clear and the choice of animal model is justified.

As the origins of Wormian bones is important, it may be useful to cite the literature demonstrating that its prevalence exhibits genetic variation. Ie: the prevalence may be higher in some populations. This is eluded to in text, but can be stated directly.

The difference across populations has been highlighted (L51-L53) and the following sentence has been added to L56: "It is clear that the incidence of Wormian bones varies across populations and this may be an indication that its prevalence exhibits genetic variation."

Line 99: Add is after hypothesis.
Added.

Lines 138-139 : change was to were. Wormian bones is plural.
Changed.

Line 320: remove the before BMD
Removed.

UCD School of Mechanical and Materials Engineering
Scoil na hInnealtóireachta Meicniúla agus Ábhar UCD

University College Dublin,
Belfield, Dublin 4, Ireland
An Coláiste Ollscoile Baile Átha Cliath,
Belfield, Baile Átha Cliath 4, Eire

T +353 1 716 1889
F +353 1 283 0534
aisling.niannaidh@ucd.ie
www.ucd.ie/eacollege/mme/

Line 336 "By eliminating the geometric limitations associated with testing irregular cranial bone samples in 3 point bending, it was proven" would read better as "Eliminating the geometric limitations associated with testing irregular cranial bone samples supported our hypothesis that…"
Changed.

Line 359: stiffness should not be capitalized.
Changed.

In the conclusion or introduction, I would appreciate a few sentences on the potential significance of the authors hypothesis. What contribution will these findings make to the field of skeletal biology? It seems to me that analyzing the biomechanical contribution of Wormian bones offers new insight into their evolution and prevalence across vertebrate species/ populations. A brief discussion of how this research advances the field would improve the article.

An additional paragraph (L98-L108) has been added to the introduction incorporating some of the points and references outlined by reviewer 1. Additionally, 2 sentences have been added to the end of the discussion (L367-371) and to the conclusion (L392-396) to highlight the potential significance of our findings.

In general, the figures are well presented and easy to understand. It may be helpful to present individual data points in box-plots, but this is not necessary.

Kind regards,

Dr. Aisling Ní Annaidh
Associate Professor
School of Mechanical and Materials Engineering
University College Dublin
